# Long-Term Humoral Immune Dynamics in Healthcare Workers with Hybrid and Vaccine-Induced Immunity: The VaCoMRI Study

**DOI:** 10.3390/v18091040

**Published:** 2026-09-19

**Authors:** Mehmet Tekinsoy, Osman Merdan, Rabia Rusen, Catharina Christa, Katharina Müller, Alina Priller, Hrvoje Mijocevic, Hedwig Roggendorf, Otto Zelger, Kathrin Tinnefeld, Samuel D. Jeske, Duluur Vasilev, Sarah Yazici, Johanna Erber, Dieter Hoffmann, Percy A. Knolle, Ulrike Protzer

**Affiliations:** 1 Institute of Virology, School of Medicine and Health, Technical University of Munich, 81675 Munich, Germany; osman.merdan@tum.de (O.M.); protzer@tum.de (U.P.); 2 Institute of Molecular Immunology, University Hospital Rechts der Isar, Technical University of Munich, 81675 Munich, Germany; 3 Occupational Medicine, University Hospital Rechts der Isar, Technical University of Munich, 81675 Munich, Germany; 4 Department for Internal Medicine II, University Hospital Rechts der Isar, Technical University of Munich, 81675 Munich, Germany; 5 German Center for Infection Research (DZIF), 81675 Munich, Germany; 6 Helmholtz Center Munich, 81675 Munich, Germany

**Keywords:** SARS-CoV-2, healthcare workers, BNT162b2, antibody kinetics, anti-spike IgG, anti-nucleocapsid IgG, surrogate virus neutralization, breakthrough infection

## Abstract

Objectives: The VaCoMRI study tracked a cohort of healthcare workers from 2020 through June 2024 to characterize the long-term sustainability of hybrid and vaccine-induced SARS-CoV-2 immunity. Methods: An initial anti-nucleocapsid (N) IgG screening of 4554 health-care workers in early 2020 identified participants who had been infected during the first wave. Anti-N-positive and anti-N-negative individuals then received BNT162b2 through the institutional vaccination program, yielding groups with hybrid and vaccine-induced immunity. Both groups were monitored regularly for nearly four years for breakthrough infections (BTI). Serum collected regularly after vaccination and after BTI was analyzed for anti-N, quantitative anti-spike (anti-S) IgG, and surrogate viral neutralizing antibody (sVNT) titers. Results: Over a median follow-up of 1180 days, 142 healthcare workers contributed longitudinal samples; 66.2% experienced one BTI and 14.1% a second. In initially seronegative individuals, anti-N titers decreased 2.3-fold between 3 and 6 months after the first BTI, with seropositivity dropping from 82% to 40.4% (median time to seronegativity: 179 days). Prior anti-N seropositivity was associated with 4.5-fold higher anti-N IgG levels after BTI, with elevated levels persisting through 9 months. In both groups, the third vaccine dose significantly enhanced anti-S antibody persistence after 9 months compared to the second dose. In infection-naïve individuals, anti-S IgG titers remained 4.2-fold and sVNT titers 7.2-fold higher. Hybrid immunity was similarly durable: two vaccine doses after prior anti-N seropositivity yielded anti-S IgG and sVNT levels comparable to three-dose vaccination. Conclusions: This study demonstrates a rapid waning of anti-N IgG that severely limits its reliability for retrospective serosurveillance of SARS-CoV-2 exposure in occupational settings. The combination of vaccination and infection induced robust and durable spike-directed antibody responses. However, these were determined using assays based on the ancestral spike protein. This may limit the prediction of protection from SARS-CoV-2 variants, which usually cause BTIs.

## 1. Introduction

Severe Acute Respiratory Syndrome Coronavirus 2 (SARS-CoV-2) emerged in Wuhan, China, in late 2019, spreading worldwide and being declared a pandemic in March 2020. By May 2026, the World Health Organization (WHO) had recorded over 7.1 million reported Coronavirus Disease 2019 (COVID-19) deaths globally [1]. In Germany, both the country’s first confirmed COVID-19 case and its first detected Omicron cases were reported in the Munich/Bavaria region [2,3]. The introduction of mRNA vaccines in late 2020 reduced the population risk of severe disease and hospitalization, transforming pandemic control in healthcare and community settings [4]. Widespread vaccination campaigns and recurrent waves of infection have created complex layers of immunity across populations. While mRNA vaccines effectively trigger antibodies against the viral spike protein, their levels naturally decline, as demonstrated by multiple longitudinal cohorts [5,6].

SARS-CoV-2 vaccination elicits spike-directed neutralizing responses, which are shaped by prior infection and booster exposures. Longitudinal cohorts have revealed anti-nucleocapsid (N) IgG declines substantially faster than anti-spike (S) IgG, often resulting in seroreversion. In contrast, S-directed binding and neutralizing activities are more durable, particularly following a booster [7]. In Germany, the vaccination campaign began in December 2020, with healthcare workers among the first priority groups. Two doses of BNT162b2 were given three weeks apart, and a third dose was recommended from late 2021. Fourth and further doses followed contemporaneous national recommendations for at-risk and occupationally exposed groups.

Multi-year antibody dynamics inform HCW protection and booster planning. Although mRNA vaccines induce robust initial immunity, long-term studies spanning repeated exposures over several years are limited. Additionally, the rapid decay of anti-N IgG complicates retrospective confirmation of prior infections.

To address these issues, we established the VaCoMRI study (SARS-CoV-2 Vaccination-Induced Immune Response in Employees of the TUM University Hospital rechts der Isar, Munich) [8], a longitudinal cohort of hospital employees that built on the pre-vaccination serostatus from the prior serology screening study “SeCoMRI” (Seroconversion to SARS-CoV-2 in Employees of the TUM University Hospital rechts der Isar, Munich) [9]. We closely monitored vaccination and breakthrough infection (BTI) events and focused on interpretable serologic endpoints: anti-N IgG (prior infection), surrogate neutralization, and anti-S IgG.

## 2. Methods

### 2.1. Study Design, Participants, and Follow-Up

During the SeCoMRI study in April–May 2020, 4554 hospital employees were screened for anti-N IgG. Starting in December 2020, SeCoMRI participants were invited to enroll in the longitudinal VaCoMRI study with follow-up visits scheduled after each vaccination and after a BTI.

We included HCWs who had received at least 3 vaccine doses and contributed repeated samples through June 2022, with additional follow-up visits captured through June 2024, where available. Prior anti-N seropositivity is defined as a positive anti-N IgG measurement at any given time point before the documented BTI. BTI was defined as an infection confirmed by positive SARS-CoV-2 testing after three vaccine doses. Hybrid immunity refers to vaccinated participants who were seropositive for anti-N before vaccination. Vaccine-induced immunity refers to participants with neither anti-N seropositivity nor a documented infection at any point before vaccination, whose responses are therefore derived solely from vaccination.

### 2.2. Data Collection and Serological Assays

Detailed vaccination and infection histories were recorded at each visit. Serum samples were collected at standardized time windows following each index event (vaccination or BTI).

To evaluate immune responses, we measured anti-N IgG (iFlash, YHLO; positive ≥ 10 AU/mL) (primarily to determine prior infection), anti-S IgG (Architect, Abbott; positive ≥ 50 AU/mL, upper limit of quantification (ULOQ) = 40,000 AU/mL), and surrogate viral neutralizing antibody (sVNT) titers (iFlash, YHLO; positive ≥ 10 AU/mL, ULOQ = 800 AU/mL).

### 2.3. Ethics

Both study protocols, including participant information, informed consent form, and informational material to promote the study, were approved by the local ethics committee of the Technical University of Munich (ethics vote 476/20 (SeCoMRI) and 26/21S-SR (VaCoMRI)) and conducted in accordance with the Declaration of Helsinki; written informed consent was obtained from all participants.

### 2.4. Statistical Analysis

We evaluated longitudinal antibody kinetics and surrogate neutralization dynamics using linear and Bayesian mixed-effects regression models. The models incorporated fixed effects (e.g., age, sex, measurement timepoint, and prior seropositivity) and participant-level random intercepts to account for repeated measurements.

Further details regarding specific sampling schedules, event handling, assay principles, and statistical modeling parameters can be found in the Supplementary Methods.

## 3. Results

### 3.1. Cohort and Follow-Up

We analyzed 142 hospital employees, of whom 94 (66.2%) were female. In 2021, the mean age was 42.8 years with a standard deviation (SD) of 12.4 years. Median age was 44 years in females and 36 years in males. Overall, 65/142 (45.7%) participants were <40 years old, 61/142 (43.0%) were 40–59 years old, and 16/142 (11.3%) were ≥60 years old.

**Figure 1:**
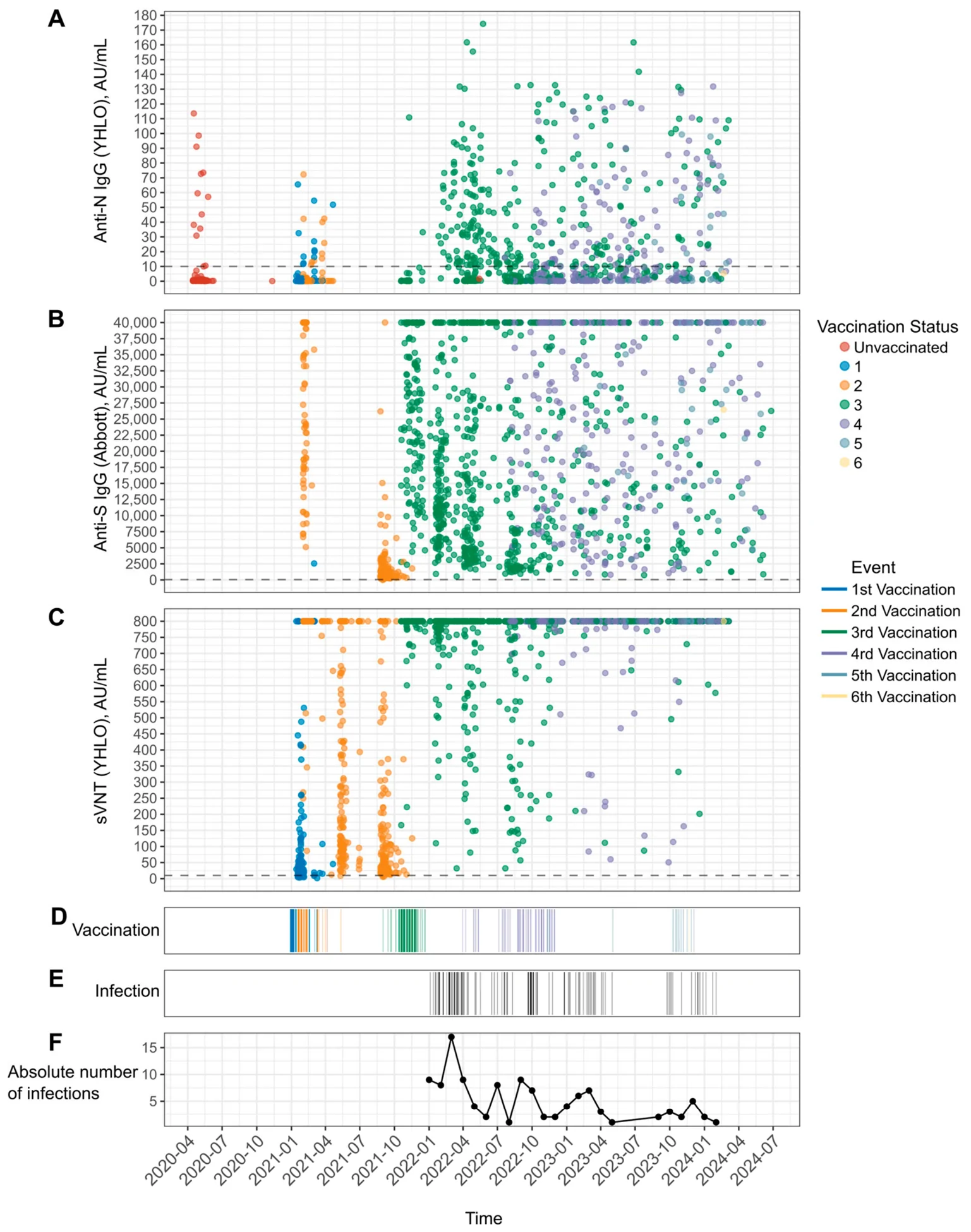
Overview of all data points collected throughout the study. Individual measurements of (**A**) anti-N IgG, (**B**) anti-S IgG, and (**C**) surrogate virus neutralization (sVNT) titers are shown, with colors indicating participants’ vaccination status at the time of sampling. Each dot represents an individual measurement, and dashed horizontal lines indicate the assay-specific positivity thresholds. (**D**) Vaccination and (**E**) infection events are indicated by vertical lines. (**F**) Total number of SARS-CoV-2 infections detected per month during the study.

Participants contributed 4102 serological measurements across 1775 study visits, averaging 12.5 per participant. Figure 1A–C provides an overview of all serological measurements (anti-N IgG, anti-S IgG, and sVNT) across the study period. The median follow-up from first contact to last visit was 1180 days.

By the end of follow-up, most participants had received three vaccine doses, with one participant receiving up to six vaccine doses: three doses in 77/142 (54.2%), four doses in 51/142 (35.9%), five doses in 13/142 (9.2%), and six doses in 1/142 (0.7%) (Figure 1D). The interval between the first and second doses was tightly clustered (median 21 days, IQR 21–22). The median time from the second dose to the third dose was 279 days (IQR: 268–289). Among those who received additional booster vaccinations, the median interval to dose 4 was 321 days (IQR 278–364; *n* = 65) and that to dose 5 was 370 days (IQR 354–441; *n* = 14).

### 3.2. Breakthrough Infections (BTIs)

One BTI (first BTI) was documented in 94/142 (66.2%) participants, with a second BTI in 20/142 (14.1%). Figure 1E,F illustrate the temporal distribution of BTIs. At the time of the first BTI, most individuals had received three vaccine doses (74/94, 78.7%); 17/94 (18.1%) participants had received four vaccine doses, and 3/94 received (3.2%) five doses. The time span from the last vaccination to the first BTI was variable, with a median of 144 days (range 28–683). At second BTI, 14/20 (70.0%) participants had received three vaccine doses, and 6/20 (30.0%) had received four doses, with a median time since last vaccination of 432 days (range 26–771). The median interval between first and second BTI was 307 days (range 107–696).

Among those who experienced the first BTI, 12/94 participants had evidence of prior anti-N seropositivity resulting from exposure to the virus.

### 3.3. Anti-N IgG Dynamics

**Figure 2:**
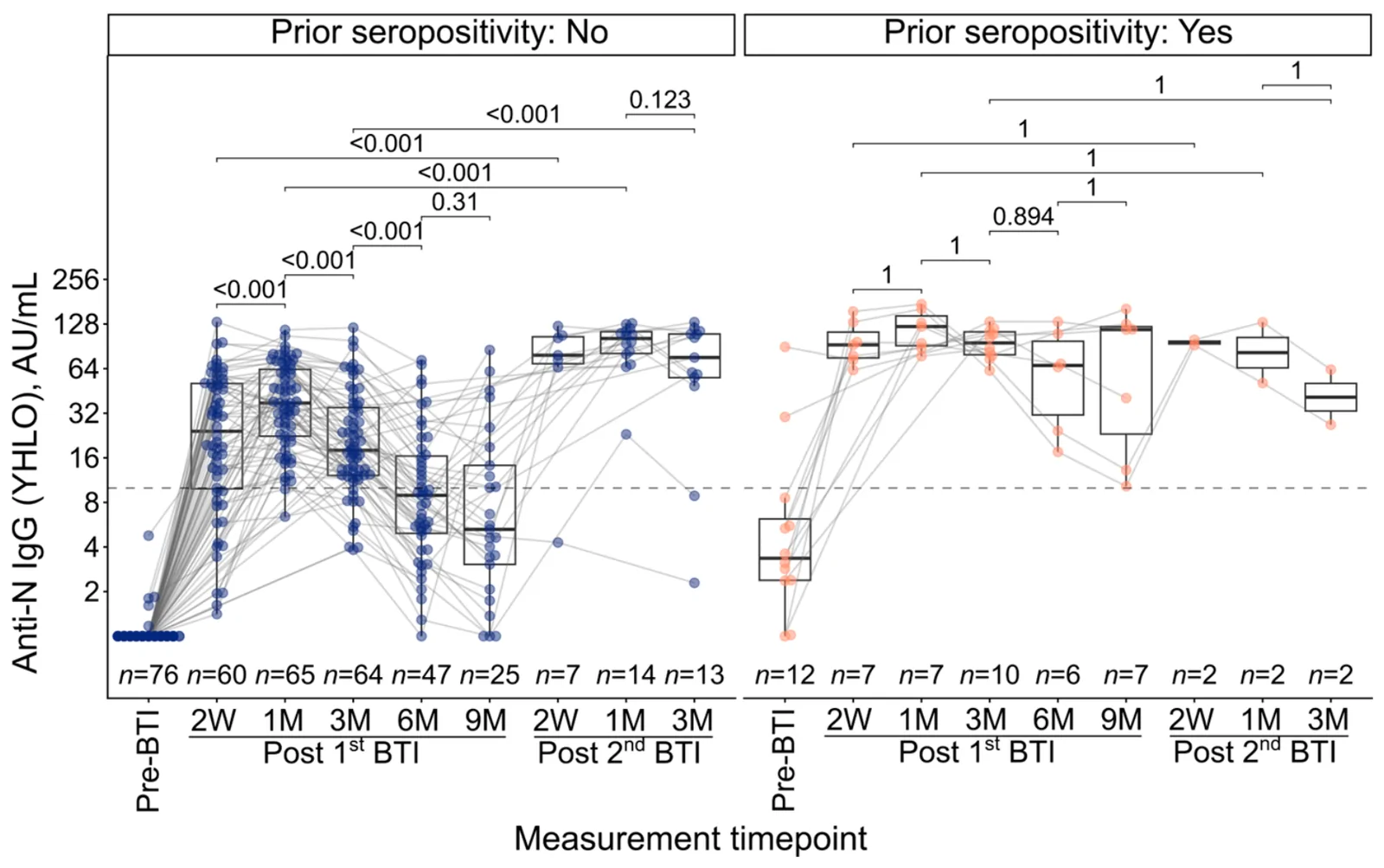
Temporal dynamics of anti-N IgG titers after first and second BTIs. The study group was divided into seropositive and seronegative subgroups based on prior anti-N IgG serostatus, indicating exposure to SARS-CoV-2 before study inclusion. Left panel: no prior anti-N seropositivity; right panel: seropositivity for anti-N. Dots represent individual measurements. Measurements from each subject are connected with lines. Statistical analysis was performed using a linear mixed-effects model with a random intercept for participants. Comparisons between individual time points were made using estimated marginal means derived from the fitted random-intercept linear model, treating the measurement time point as a categorical variable. Holm-adjusted *p*-values for the corresponding planned comparisons are displayed. *Y*-axis values were log 2-scaled. For each measurement time point, the number of available samples (*n*) is shown. Horizontal dashed lines indicate the threshold value (10 AU/mL) for a positive test result. Pre-BTI marks the most recent available measurement before the detection of the first BTI. AU: Arbitrary units. W: Week(s); M: Month(s), BTI: Breakthrough infection.

In total, 1225 anti-N IgG measurements were available (Figure 1A). Following the first and second BTIs, anti-N titers showed progressive waning with substantial inter-individual variability (Figure 2).

Anti-N IgG titers in participants without prior anti-N seropositivity peaked between 2 and 4 weeks and declined between 1 and 6 months, with a significant boosting effect after the second BTI (Figure 2). Age and sex were not significantly associated with anti-N IgG levels (*p*-values: 0.74 and 0.44, respectively). Individuals with prior anti-N seropositivity had estimated 4.5-fold higher anti-N IgG levels (95% CI: 2.5–8.3, *p* < 0.001), showing a booster effect upon repeat exposure.

Among participants without prior infection, model-based estimates indicated a 1.8-fold (95% CI: 1.3–2.4; Holm-adjusted *p* < 0.001) decrease between 1 and 3 months after the first BTI, and a 2.3-fold (95% CI: 1.6–3.1; Holm-adjusted *p* < 0.001) decrease between 3 and 6 months. Parallel to titers, the seropositivity rate declined between post-first BTI at 3 months (82%, 53/64) and 6 months (40.4%, 19/47). Overall, the first negative anti-N after the first BTI was recorded at a median of 179 days (*n* = 54, IQR = 15–239).

One month after the second BTI, titers were estimated to be 2.6-fold (95% CI: 1.5–4.3; Holm-adjusted *p* < 0.001) higher than the same time point after the first BTI. Comparison of titers three months after second BTI versus first BTI revealed approximately a 3-fold increase (95% CI: 1.72–5.12; Holm-adjusted *p* < 0.001).

### 3.4. Anti-Spike IgG

**Figure 3:**
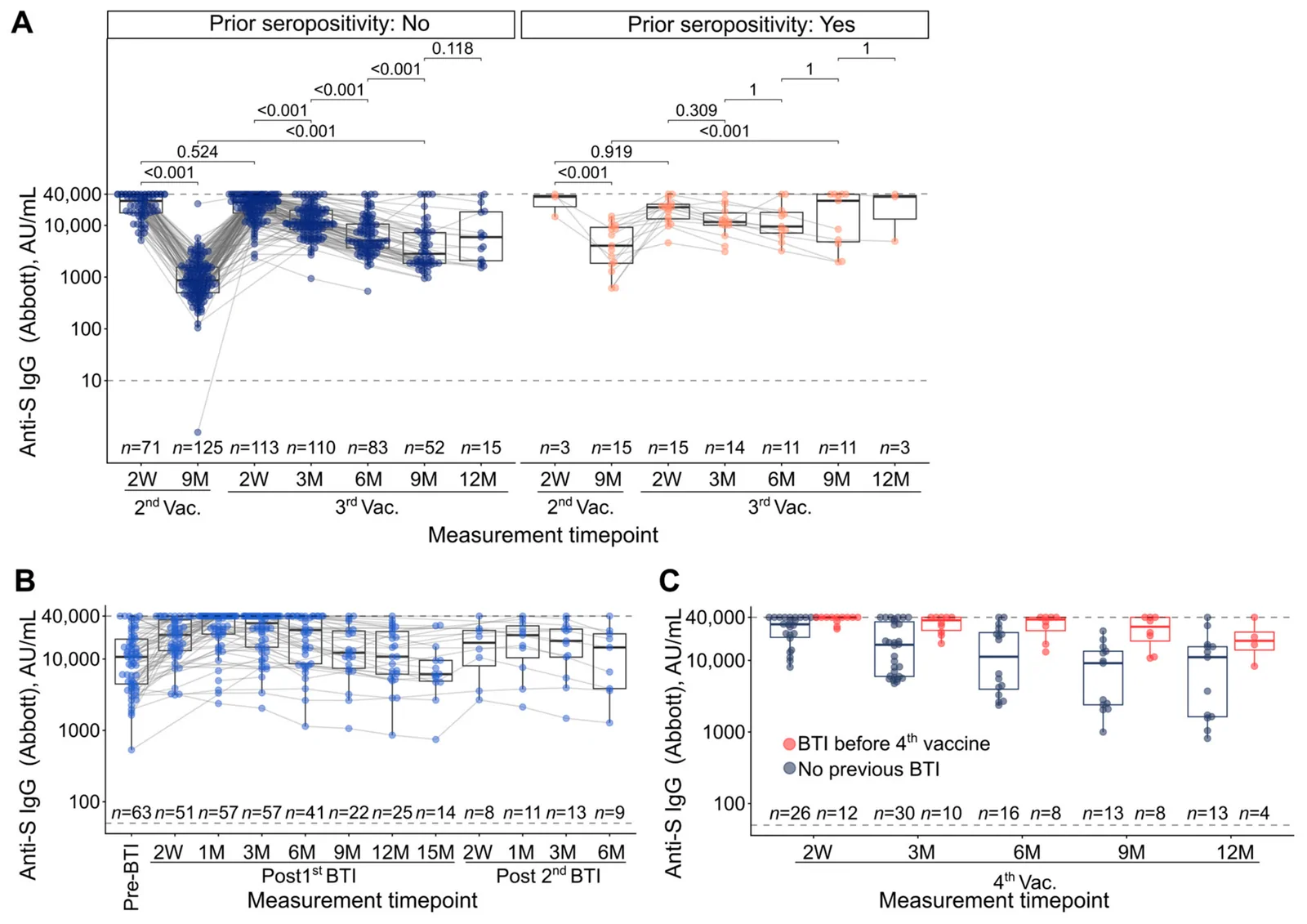
Temporal dynamics of anti-S IgG titers (**A**). Change in anti-S IgG titers after 2nd and 3rd vaccination doses, factored for prior seropositivity of anti-N IgG representing prior exposure to SARS-CoV-2, is presented. For selected time points, all available measurements before any BTI are shown. Dots represent individual measurements. Measurements from each subject are connected with lines. Statistical analysis was performed using a linear mixed-effects model. Comparisons were made using estimated marginal means derived from the fitted random-intercept linear model, treating the measurement time point as a categorical variable. Holm-adjusted *p*-values for the corresponding planned comparisons are displayed. (**B**). Anti-S IgG titer traces of post-BTI measurements among participants who received three vaccinations. Pre-BTI measurements are the most recent measurements taken before the BTI, which are no more than 120 days old. (**C**). Summary of the measurements taken after the 4th vaccination before a new BTI. Measurements are colored based on the previous BTI history after the 3rd vaccination. *Y*-axis values were log 10-scaled. For each measurement time point, the number of available samples (*n*) was shown. Horizontal dashed lines indicate the threshold value (50 AU/mL) for a positive test result, and the assay upper limit of quantitation (40,000 AU/mL). W: Week(s); M: Month(s); Vac: Vaccination.

In total, 1293 quantitative anti-S IgG measurements were collected (Figure 1B). To compare S antibody kinetics after the second and third doses, only measurements obtained before any BTI were included (Figure 3A).

In participants without prior anti-N seropositivity, anti-S IgG titers declined significantly between 2 weeks and 9 months after the second dose, with an estimated 27.5-fold reduction (95% CI: 21.3–34.7; Holm-adjusted *p* < 0.001) (Figure 3A). Because 32.4% (24/74) of measurements at 2 weeks post-second-dose were at the assay ULOQ, the resulting ceiling effect may have limited discrimination of peak antibody responses and influenced the magnitude of the calculated fold-change. A third booster dose restored antibody titers to a similar level 2 weeks after the second vaccination (Holm-adjusted *p* = 0.638) (Figure 3A). Two weeks after the third vaccination, 18.8% (25/133) of measurements were at the assay ULOQ (anti-S IgG titer IQR: 17,325–37,486). Compared to the second dose, a slower waning of titers was observed after the third dose, consistent with observations of sVNT titers. Model-based estimates following the third vaccine dose from 2 weeks to 3 months, 3 to 6 months and 6 to 9 months showed 1.89-fold (95% CI: 1.52–2.35; Holm-adjusted *p* < 0.001), 1.9-fold (95% CI: 1.53–2.46; Holm-adjusted *p* < 0.001) and 1.6-fold (95% CI: 1.2–2.3; Holm-adjusted *p* < 0.001) decreases respectively. Notably, at the 9-month mark, titers remained 4.2-fold higher following the third dose compared to the second dose (95% CI: 3.2–5.6; Holm-adjusted *p* < 0.001) (Figure 3A).

Prior anti-N seropositivity and sex were not statistically significant predictors of antibody titer (*p* = 0.23 and 0.24, respectively). However, prior anti-N seropositivity and measurement timepoint interaction effects were significant at 9 months after the second vaccination, implying almost 6-fold higher titers (*p* = 0.0018). Age was a relevant factor (*p* = 0.0083); a 1 SD (12.4 years) increase in age was associated with a 13% decrease (95% CI: 4–23%) in titer. To compare hybrid immunity with booster vaccination, we contrasted participants with prior anti-N seropositivity who had received two vaccine doses with infection-naïve participants who had received three doses, in both cases 9 months after the last dose and before any BTI (Figure 3A). Median anti-S IgG titers were 4054 AU/mL (IQR 1854–9191) and 2826 AU/mL (IQR 1845–7258), respectively. Given the small number of participants with hybrid immunity and a ceiling effect at the assay ULOQ, these data are reported descriptively and were not formally tested.

After the first BTI in triple-vaccinated participants, anti-S IgG titers peaked around 1 month, when 50.9% (29/57) of measurements were at the assay ULOQ (IQR: 22,319–40,000) (Figure 3B). After 12–15 months, values approached those before BTI levels.

All measurements after the third vaccination were above the assay-positive threshold. After the fourth vaccination, anti-S IgG measurements were scarce (Figure 3C). Participants were divided into two groups: one with BTI before the fourth vaccination and the other with no prior documented infection. Exploratory analysis showed a trend toward slower antibody titer decline among participants with prior BTI.

### 3.5. Surrogate Viral Neutralizing Antibody Dynamics

In total, 1584 sVNT measurements were available after vaccination (Figure 1C). To evaluate longitudinal vaccine-induced immunity before any BTI, we analyzed measurements from infection-free participants 2 weeks post-first dose, 3 and 9 months post-second dose, and 6 and 9 months post-third dose.

**Figure 4:**
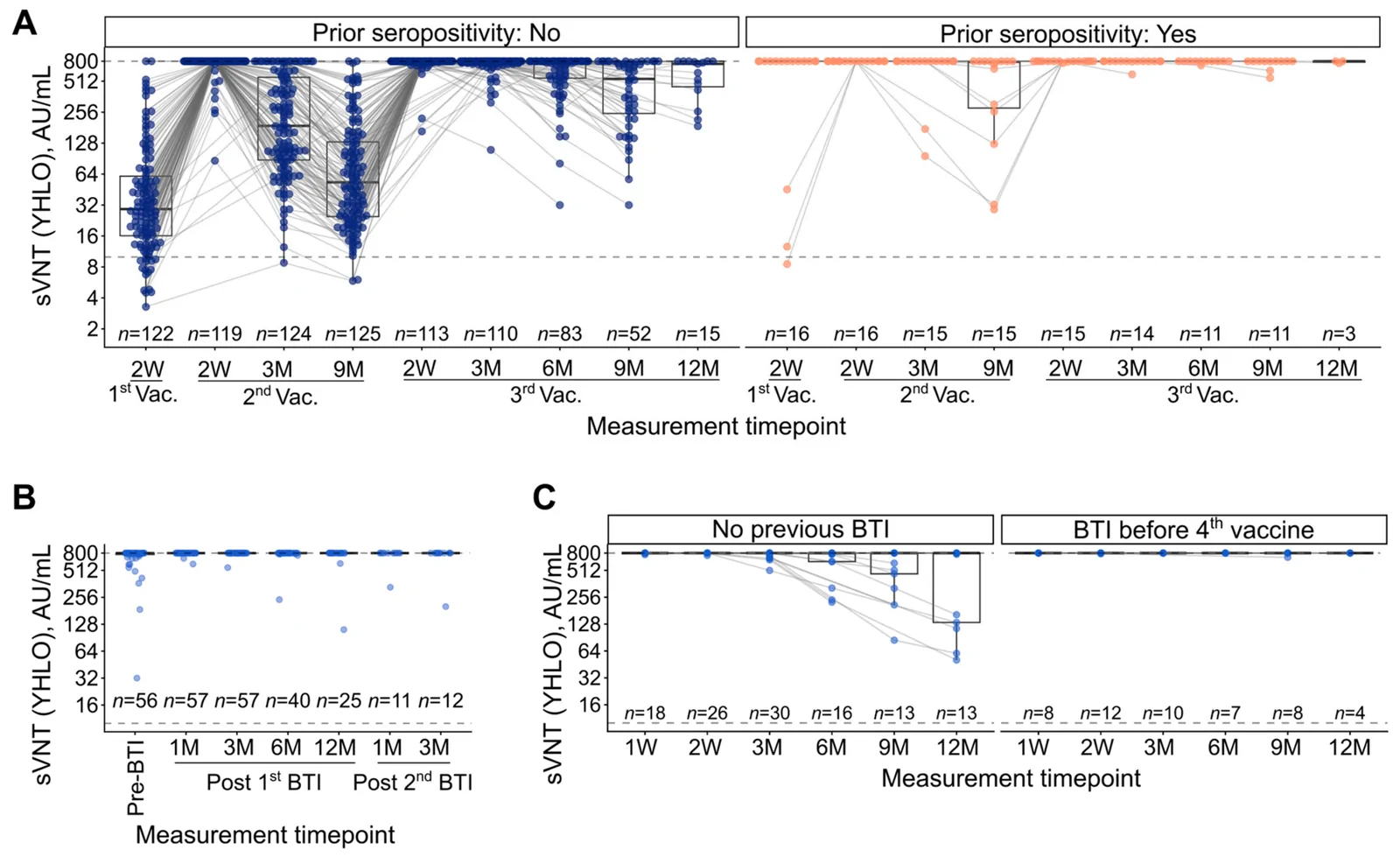
Surrogate virus neutralization (sVNT) titers after vaccination and BTI. (**A**) The study group was divided by prior seropositivity for anti-N IgG, indicating prior exposure to SARS-CoV-2. Left panel: no prior anti-N seropositivity; right panel: seropositivity for anti-N. sVNT titers (YHLO, AU/mL) over time in study participants without documented breakthrough infection are displayed. (**B**) sVNT titers over time among participants with first and second BTI after 3rd vaccination. Pre-BTI measurements are the most recent measurements taken before the BTI, which are no more than 90 days old. (**C**) Summary of the measurements taken after the 4th vaccination before a new BTI. Measurements are facetted based on the previous BTI history after the 3rd vaccination. *Y*-axis values were log 10-scaled. For each measurement time point, the number of available samples (*n*) is shown. Horizontal dashed lines indicate the threshold value (10 AU/mL) for a positive test result, and the assay upper limit of quantitation (800 AU/mL). W: Week(s); M: Month(s); Vac: Vaccination.

Two weeks after the first vaccination, the median sVNT titers were 29.2 (*n* = 122, IQR: 16.1–61.1) and 800 AU/mL (*n* = 16) in participants without and with prior anti-N seropositivity, respectively (Figure 4A). Following the second vaccination, almost all measurements reached the assay ULOQ (Figure 4A), resulting in a pronounced ceiling effect that limited discrimination of peak neutralizing responses and may have influenced the magnitude of the subsequent fold-change estimates. Three months later, the median titer dropped to 188 AU/mL among participants without prior anti-N seropositivity. In this group, a mean 3.5-fold decline (95% CrI: 3.1–3.8) was observed between 3 and 9 months; titers at 9 months were 1.7-fold (95% CrI: 1.46–1.9) higher than at 2 weeks post-first vaccination (Figure 4A).

The third dose provided a much larger boost than the second. sVNT titers remained at or very close to assay ULOQ for at least 3 months (Figure 4A). Despite a significant 2.5-fold (95% CrI: 1.7–3.7) decay being observed between 6 and 9 months, antibody levels 9 months after the third vaccine dose were 7.2-fold higher (95% CrI: 5.4–9.5) than those recorded after the second dose. Overall, after the second vaccination dose, except for three measurements, all analyzed measurements exceeded the assay-positive threshold.

Prior anti-N seropositivity strongly predicted higher responses, yielding 8-fold higher titers (CrI: 3.1–23.5) nine months post-third vaccination. A 1-SD increase in age (12.4 years) reduced titers by 39% (CrI: 26–48%), while sex effects were negligible. Participants with prior anti-N seropositivity who had received two vaccine doses were also compared with infection-naïve participants who had received three doses for surrogate neutralization, again 9 months after the last dose and before any BTI (Figure 4A). Median sVNT titers were 790 AU/mL (IQR 281–800) and 539 AU/mL (IQR 249–782), respectively. Given the small number of participants with hybrid immunity and a pronounced ceiling effect at the assay ULOQ, these data are reported descriptively.

sVNT titers after BTIs post-third dose were consistently at or very close to the ULOQ. Six months after the first BTI, 82.5% of sVNT measurements (33/40) were at ULOQ. Similarly, 12 months after the first BTI, 88% of sVNT measurements (22/25) were at ULOQ.

Parallel to observations of post-third-vaccination titers among BTI-free individuals, neutralizing antibody titers remained at or near the assay ULOQ after the fourth vaccination.

However, some individuals showed a visible decay in sVNT titers after 3 months. Although measurements were limited for individuals who experienced a BTI between the third and fourth vaccine doses, the available data showed that their titers remained consistently at the ULOQ.

## 4. Discussion

In this longitudinal cohort of 142 hospital employees followed for nearly four years, we observed contrasting dynamics between vaccine-induced and infection-induced humoral immunity. Repeated SARS-CoV-2 vaccination elicited robust, highly neutralizing, and sustained spike-directed antibody responses (anti-S IgG and sVNT), with a significantly slower rate of waning than after the primary vaccination series. However, persistently high spike-directed antibody levels cannot predict sterilizing immunity since both assays are based on the ancestral spike, whereas most BTIs occurred due to the Omicron VoC that can escape antibody neutralization. Thus, the measured titers reflect the persistence of the spike-directed antibody response rather than predicting protection against the infecting variants. Furthermore, unlike the durable spike-directed responses, infection-induced anti-N levels waned rapidly, with a substantial reduction in seropositivity within six months post-exposure.

A key finding of our study is the clear divergence between spike-directed and nucleocapsid-directed antibody trajectories. Following BTI, anti-N increased but then declined rapidly, resulting in a significant drop in seropositivity rates between three and six months after the initial BTI. This pattern is consistent with previous studies showing that anti-N antibodies are less persistent than spike-directed responses and often become undetectable during follow-up [7]. In contrast, both anti-S IgG and sVNT remained detectable at substantially higher levels over time, particularly after booster vaccination.

Our findings also highlight the importance of prior SARS-CoV-2 exposure in shaping subsequent humoral responses. Participants with prior anti-N seropositivity had higher anti-N IgG and sVNT levels over follow-up, and a larger proportion remained at the ULOQ for sVNT after the third dose. These results are consistent with the broader literature showing that hybrid immunity is associated with stronger and more sustained immune responses than vaccination alone. In the SIREN cohort, for example, previously infected individuals who were subsequently vaccinated maintained high levels of protection against reinfection for more than one year [10].

Our longitudinal observations of the primary vaccine series reinforce the established global consensus that mRNA vaccination triggers a robust initial humoral response, which predictably wanes over the subsequent 6–12 months [6,11,12,13,14,15]. The third vaccine dose, however, was a particularly important inflection point in our cohort. Compared with the second dose, it was associated with significantly higher anti-S IgG and sVNT responses, with many sVNT measurements reaching or approaching the assay ULOQ. This finding is consistent with published data on the immunogenicity of booster doses and with studies showing enhanced humoral responses after additional vaccine doses [16,17]. Although some measurements reached the assay ceiling, limiting discrimination of the highest responses, the overall pattern strongly supports preserved humoral recall capacity in this cohort.

Despite the strengths of our nearly four-year longitudinal follow-up and the high density of serial samples, several limitations must be acknowledged. First, the single-center design and the cohort composition, which consisted of relatively healthy hospital employees with high vaccine uptake and ongoing occupational exposure, limit generalizability. In addition, the present analysis was based on a subgroup of 142 participants who underwent repeated longitudinal sampling. This may favor individuals with greater follow-up adherence, potentially introducing selection bias. Second, serological measurements were based on commercial assays with inherent constraints. After booster vaccination, a substantial proportion of anti-S and sVNT values reached the assay ULOQ, which limits discrimination at peak response. Although the surrogate neutralization assay provides a practical approximation of neutralizing activity, it does not fully capture the complexity of live-virus neutralization. Third, infection ascertainment was heterogeneous and included institutional PCR, external PCR, and self-reported positive antigen tests. As a result, some asymptomatic or unreported infections may have been missed, and infections were not analyzed separately by diagnostic modality. In addition, because blood sampling occurred at predefined but pragmatic intervals, the precise timing of infection and serologic peak responses could not always be determined. Fourth, our study focused on humoral immunity and did not include measurements of cellular immunity. However, T-cell responses remain relevant, even when antibody levels decline. Finally, the study spanned multiple epidemiological phases, including the emergence of antigenically distinct variants, which may have influenced the apparent waning through both biologic decline and changing antigenic match over time. Importantly, both the sVNT and the anti-S assay are based on the ancestral SARS-CoV-2, whereas the majority of BTIs occurred during the Omicron era. Thus, the measured neutralizing and spike-binding antibody responses may not fully reflect activity against the SARS-CoV-2 variants responsible for infection. These measurements should therefore not be interpreted as direct indicators of protection against the infecting variants.

In this multi-year cohort of healthcare workers, repeated SARS-CoV-2 vaccination induced strong and relatively sustained spike-directed humoral responses, particularly after the third dose, whereas infection-associated anti-N IgG declined substantially faster after BTI. As anti-S and sVNT responses were measured against the ancestral spike, they describe the durability of the humoral response rather than protection against the variants that caused most BTIs in this cohort. Prior SARS-CoV-2 exposure was associated with stronger subsequent humoral responses, consistent with a hybrid immunity pattern. Ultimately, although anti-N IgG is useful for identifying recent infections, establishing multi-year or decadal serological footprints requires further research. Future research should also focus on developing alternative serological markers to comprehensively evaluate long-term protection.

## Data Availability

The study protocol for VaCoMRI is available from the authors upon reasonable request. De-identified data supporting the findings of this study can be obtained from the corresponding author upon reasonable request, subject to institutional approvals.

## Supplementary Materials

The following supporting information can be downloaded at https://www.mdpi.com/article/10.3390/v18091040/s1: Detailed Methods.
