# Supplementary material for: Long-Term Humoral Immune Dynamics in Healthcare Workers with Hybrid and Vaccine-Induced Immunity: The VaCoMRI Study
Source: Viruses. 2026 Sep 19;18(9):1040. doi: 10.3390/v18091040 (PMC13611903; doi:10.3390/v18091040)
Supplement: Supplementary file 1 [file viruses-18-01040-s001.zip › viruses-4479708-supplementary.pdf]

Supplementary Methods

Study design, participants, and follow-up

At the SeCoMRI baseline study in April–May 2020, 4,554 hospital employees were screened for anti-N IgG antibodies using the iFlash platform (see below). From December 2020 onward, SeCoMRI participants were invited to longitudinal follow-up aligned with the institutional vaccination rollout in the VaCoMRI study.

VaCoMRI participants were followed up after each vaccination and after BTI. By June 2024, population-level vaccine uptake in Germany was high, with 76.4% having completed two vaccinations and 62.6% having received at least a third vaccine dose. Against this background, and to ensure contemporary relevance in a highly immunized setting, we included HCWs who had received at least 3 vaccine doses and contributed repeated samples through June 2022, with additional follow-up visits captured through June 2024, where available.

Eligibility

Eligible participants were adult employees of University Hospital rechts der Isar who had participated in the SeCoMRI study and provided written informed consent for recontacting and reconsenting, repeated venous blood sampling, biobanking, and the use of study-related data. Participants were excluded if they withdrew consent or if repeated sample collection was not feasible. Documented conditions expected to substantially affect immune function, when known, were considered during cohort selection and interpretation.

Ethics

Both study protocols, including participant information, informed consent form, and information material to promote the study, were approved by the local ethics committee of the Technical University of Munich (ethics vote 476/20 (SeCoMRI) and 26/21S-SR (VaCoMRI)) and conducted in accordance with the Declaration of Helsinki; written informed consent was obtained from all participants.

Data collected

Demographics (age and sex) and detailed vaccination and infection histories were recorded at each visit.

Vaccination exposure

Participants received BNT162b2 mRNA for the first three doses per the institutional program. Subsequent vaccinations (fourth, fifth, and sixth doses, where applicable) followed

contemporaneous public health recommendations; their timing relative to blood draws was captured using the visit windows described below.

Definitions of index events

Vaccination events were defined by the first and all subsequent SARS-CoV-2 vaccine doses. BTI was described as a SARS-CoV-2 infection occurring after any vaccine dose, documented in clinical records or reported to the study. Baseline was defined as a sample obtained before the first vaccine dose.

Prior anti-N seropositivity is defined as a positive anti-N IgG measurement at any given time point before the documented BTI.

Infection ascertainment

SARS-CoV-2 infections were ascertained by either a documented positive PCR result (performed at our institute or an external laboratory) or self-reported positive antigen tests conducted by participants.

Sampling schedule

At each index event, samples were targeted at standardized time windows; actual draw days varied pragmatically across participants and were analyzed as observed. Samples were collected at baseline (pre-dose) and approximately 2 weeks after the first vaccine dose. After the second dose: approximately 2 weeks, 3 months (about 90–120 days), and 9 months (about 210–270 days). After the third dose (booster): approximately 2 weeks, 3 months, 6 months, and 9–12 months; in some participants, an additional 1-week sample was collected. After the fourth dose: approximately 1 week, 2 weeks, 3 months, 6 months, 9 months, and 12 months; extended follow-up included about 15 or 21 months in a subset. After the fifth dose: approximately 2 weeks, 3 months, and 6 months. After the sixth dose: approximately 3 months in a subgroup. After BTI: approximately 2 weeks and 1 month after first BTI, then about 3, 6, 9, 12, 15, 18, 21, 24, 27, 31, and 33 months when available; for participants with a second or third BTI, post-event sampling followed the same pattern (typically about 2 weeks, 1 month, 3 months, and 6–12 months after BTI).

Handling multiple events

For participants with multiple vaccines and/or BTIs, each sample was anchored to the most recent prior event. When two events occurred in close succession, samples were assigned to the event immediately preceding the draw.

Inclusion/exclusion of visits and adherence

All available time points listed above were included; missed visits were not imputed. Because windows were pragmatic, analyses used the observed day-after-event values rather than enforcing nominal visit days.

Specimen collection and processing

Peripheral blood was collected at each visit and processed using standard procedures for serum isolation. Assay-specific laboratory methods are detailed in the subsection on serological assays.

Serological assays

Anti-nucleocapsid (anti-N) IgG (iFlash, YHLO)

Serum was tested for anti-SARS-CoV-2 nucleocapsid IgG using a chemiluminescent immunoassay (CLIA) on the iFlash analyzer (YHLO Biotech, Shenzhen, China), following the manufacturer's instructions. Results were reported as assay units (AU) and interpreted using a prespecified cut-off: anti-N IgG ≥ 10 AU was considered positive; values < 10 AU were considered negative. This assay was primarily used to infer prior infection at baseline and during follow-up, as indicated.

Anti-spike (anti-S) IgG (Architect, Abbott)

Antibodies binding the SARS-CoV-2 spike receptor-binding domain (RBD) IgG were measured using the Abbott IgG II Quant CMIA on the Architect platform per the manufacturer's instructions. Results were expressed in arbitrary units per milliliter (AU/mL). Seropositivity was defined as anti-S IgG ≥ 50 AU/mL.

Surrogate Viral Neutralizing Antibody titers (sVNT) (iFlash, YHLO)

Serum neutralizing activity was determined using the iFlash SARS-CoV-2 Neutralizing Antibody assay (YHLO Biotech) on an iFlash 3000 instrument, following manufacturer instructions. The assay determines the surrogate neutralization capacity of the SARS-CoV-2 Wuhan strain using a competitive CLIA. First, patient serum is mixed with RBD-coated magnetic particles, allowing anti-SARS-CoV-2 antibodies to bind the recombinant RBD expressed in mammalian cells. Next, acridinium-labeled ACE2 is added and detected by chemiluminescence; it binds only RBD moieties that were not already occupied by antibodies. Because of this competition, a weaker light signal means stronger neutralization. To report these results intuitively, the instrument's software automatically converts the relative light units (RLUs) into AU/mL using a master calibration curve. In this inverted calculation, higher AU/mL values directly correspond to higher neutralization capacity. Results were reported in AU/mL, with ≥10 AU/mL predefined as positive, representing the surrogate virus neutralization titer (sVNT).

Outcomes

Primary outcomes were immune readouts measured at each scheduled time point relative to the triggering event (vaccination or BTI). Secondary outcomes included durability, measured

as change over months after each event, and comparative responses across events (e.g., dose 3 versus BTI).

Statistical analysis

R statistical software (version 4.5.1) and packages were used for all statistical analyses. Plots were created using the ggplot2 package (4.0.3).

Kinetics of anti-N IgG titers on a temporal scale were investigated with a linear mixed-effects regression model using the lmer function from the lme4 package in R (version 2.0). The model was fitted with log2 IgG levels as the dependent variable, using measurements at 2 weeks, 1 month, 3 months, and 6 months after the 1st BTI, and at 2 weeks, 1 month, and 3 months after the 2nd BTI. All available measurements were used. Fixed effects comprised standardized age (mean: 41.2, sd:12.1), sex, measurement period (categorical), prior anti-N IgG seropositivity status, and the interaction between measurement period and prior seropositivity status. A random intercept for participant identifier was specified to model between-subject variability and account for repeated measures. Estimation was performed using restricted maximum likelihood (REML). P-values for fixed effects were obtained using the lmerTest package (version 3.2), with degrees of freedom approximated using Satterthwaite's method. Confidence intervals for fixed-effect estimates were obtained using bootstrap resampling. Post hoc comparisons were based on estimated marginal means from the linear mixed-effects model, using the emmeans package (2.0.3). Custom contrasts were defined a priori to assess longitudinal changes within measurement periods and differences between first and second BTI at matching time points. Multiple testing correction was applied using the Holm method.

Anti-S antibody titer kinetics before any BTI infection were investigated using a linear mixed-effects regression model in lmer from the lme4 package. The model was fitted with log10 IgG levels as the dependent variable, using all available measurements at 2 weeks and 9 months post-2nd vaccination, and at 2 weeks, 3 months, 6 months, 9 months and 12 months post-3rd vaccination. Fixed effects comprised standardized age (mean: 42.8, sd: 12.4), sex, measurement timepoint (categorical), prior anti-N IgG seropositivity status, and the interaction between measurement period and prior anti-N IgG seropositivity status. A random intercept for participant identifier was specified to model between-subject variability and account for repeated measures. Estimation was performed using restricted maximum likelihood (REML). Initially, a model without an interaction term was fitted and compared to a model with the interaction term using the conditional Akaike information criterion (cAIC). The model with prior seropositivity and measurement timepoint interaction yielded a lower cAIC score, indicating a better fit, and was selected for further investigation. P-values for fixed effects were obtained using the lmerTest package with Satterthwaite's method, and

confidence intervals were derived from bootstrap resampling. Post hoc comparisons were based on estimated marginal means from the linear mixed-effects model, using the emmeans package. Custom contrasts were defined a priori to assess longitudinal changes across measurement time points. Multiple testing correction was applied using the Holm method.

Longitudinal dynamics of vaccine-induced sVNT were assessed with a Bayesian linear mixed-effects model using the brms (version 2.23) framework. The dependent variable was log2-scaled and right-censored, accounting for the assay's upper limit of quantification (ULOQ = 800 AU/mL) as all measurements above this limit were reported as 800 AU/mL during sample collection and analysis. Measurements from 2 weeks post-1st vaccination, 3 and 9 months post-2nd vaccination, and 6 and 9 months post-3rd vaccination were analyzed, since more than 75% of all observations at 2 weeks post-2nd vaccination and at 2 weeks and 3 months post-3rd vaccination were at the assay ULOQ. The model included population-level fixed effects for measurement time point (categorical), prior anti-N IgG seropositivity status, the interaction between measurement time point and prior seropositivity status, age (standardized, mean = 42.8, SD = 12.4), and sex. Individual-level random intercepts were used to account for repeated measures. Weakly informative priors were used to regularize model estimates. Six chains were run on six cores with 4000 iterations and a 1000-iteration warm-up period. Model convergence was confirmed via visual inspection of trace plots and by ensuring the Gelman-Rubin statistic ($\hat{R}$) was 1.00 for all parameters. A prior-predictive check was conducted by simulating data from the model while ignoring the observed values to check the suitability of the chosen priors. Robustness of the Bayesian estimates to selected priors was checked by re-running the model with flat priors. Both models were compared to assess the impact of the selected priors on the final estimates. Statistical inference was based on the posterior mean and 95% Credible Intervals (CrI). Custom hypotheses were defined a priori to assess longitudinal changes within measurement periods and tested with the hypothesis function. Posterior hypothesis testing results were presented as the mean of the estimate, along with the upper and lower CrIs.
